# Racial differences in knowledge, attitudes toward vaccination, and risk practices around Lyme disease in the United States

**DOI:** 10.3389/fpubh.2025.1473304

**Published:** 2025-03-21

**Authors:** Madiha Shafquat, Niyati Patel, Brandon McFadden, James H. Stark, L. Hannah Gould

**Affiliations:** ^1^Vaccines and Antivirals Medical Affairs, Pfizer US Commercial Division, Cambridge, MA, United States; ^2^Behavioralize LLC, Wynnewood, PA, United States; ^3^Department of Agricultural Economics and Agribusiness, University of Arkansas, Fayetteville, AK, United States; ^4^Vaccines and Antivirals Medical Affairs, Pfizer US Commercial Division, New York, NY, United States

**Keywords:** health knowledge, attitudes, practices, Lyme disease, racial groups, ethnicity, health disparities

## Abstract

**Introduction:**

Lyme disease (LD) incidence in the United States is highly regional, with most cases occurring in 16 high-incidence jurisdictions. LD incidence and severity of disease have been found to vary by race. This study describes racial differences in knowledge, attitudes toward vaccination, and risk practices related to LD.

**Methods:**

Four web-based surveys were conducted with adults and caregivers of children in high-incidence jurisdictions and 10 states neighboring them. Respondents were recruited via an established online panel to represent the general population. Self-reported race was pooled into 3 categories: ‘White’, ‘Black or African American’, and ‘Other’ for analysis. Analyses were conducted separately for each jurisdiction (high-incidence vs. neighboring) and respondent type (adult vs. caregiver).

**Results:**

The final sample across all surveys included 2,249 respondents who identified as White, 493 respondents who identified as Black or African American, and 674 respondents of other races. White respondents were older, had higher incomes, and were likelier to live in small cities and rural areas. Though attitudes toward vaccination in general were similar between racial categories, when differences were present, Black respondents were more likely to have concerns about vaccines than White respondents. In all surveys, White respondents engaged in more outdoor activities than Black respondents and performed these activities more often. However, both White adults and caregivers in high-incidence jurisdictions were significantly less likely to have occupations with primarily outdoor work than corresponding respondents in other racial groups. Black respondents also had lower knowledge about LD than White respondents across all surveys. This difference was significant after adjusting for state incidence level and urbanicity.

**Conclusion:**

There are some racial differences in knowledge, attitudes, and practices around LD, with White respondents reported having higher knowledge of LD, less concerns about vaccines, and higher frequency of risk practices. These differences might contribute to racial disparities in LD outcomes.

## 1 Introduction

Lyme disease (LD) is the most common vector-borne disease in the United States, with an estimated 476,000 individuals diagnosed and treated each year (1). The causative bacterium, *Borrelia burgdorferi*, is transmitted to humans through a bite from an infected *Ixodes scapularis* tick. Clinical presentation of LD typically begins with localized disease, which often involves erythema migrans rash at the site of the tick bite. If left untreated, this can progress to more severe disseminated disease and involve neurologic, rheumatologic, or other manifestations (2). Though antibiotic therapy is effective at treating LD (2), there is no vaccine currently available to prevent infection in humans.

The incidence of LD in the United States is highly regional, as the distribution and density of infected *Ixodes* ticks are associated with ecological factors. As of 2022, the US Centers for Disease Control and Prevention (CDC) reported 16 high-incidence (incidence >10 per 100,000 population) jurisdictions where most LD cases occurred: Connecticut, District of Columbia, Delaware, Maine, Maryland, Massachusetts, Minnesota, New Hampshire, New Jersey, New York, Pennsylvania, Rhode Island, Vermont, Virginia, West Virginia, and Wisconsin (3). LD is also an emerging public health concern in jurisdictions not currently classified as high-incidence, as the areas considered at high risk of LD have spread geographically over time (4).

LD incidence is driven by entomologic risk (e.g., the presence of infected, questing *Ixodes* ticks) and human exposure to these ticks. The former necessitates that LD is a ‘disease of place’, determined by entomologic risk alone, but the latter may have differences in exposure risk resulting from knowledge and awareness of LD, attitudes toward LD risk and vaccination, and frequency of activities that put individuals in contact with questing ticks. Though the reported incidence and prevalence of LD in the US is highest in White individuals, this is likely reflective, at least in part, of the relationship between where individuals live and the presence of ticks in those environments (5). Further, though White individuals have the highest incidence rates, people from racial or ethnic minority groups are more likely to report disseminated disease and more severe outcomes (6–9). Possible explanations for this disparity in outcomes include factors such as disease recognition on darker skin tones and variations in healthcare access by race (9). However, the extent to which these differences reflect higher underlying risk due to place of residence or differences in exposure for race and ethnicity groups is unclear.

Further, there is a lack of published data on the relationship between race and knowledge, attitudes toward vaccination, and practices around LD. The limited evidence currently available has inconsistent findings on racial differences within high-incidence areas (10–15). This present study aims to address this gap using a large, representative sample across multiple jurisdictions to assess differences in these measures by race. This study includes both high-incidence jurisdictions, where efforts around LD prevention are likely to be highest, and neighboring jurisdictions where LD may be a growing public health concern due to the geographic spread of the vector.

## 2 Methods

### 2.1 Study design

Data were collected using web-based surveys distributed to respondents in jurisdictions defined as high-incidence based on CDC LD surveillance data (16) and “neighboring” states sharing a border with a high-incidence jurisdiction (i.e., Illinois, Indiana, Iowa, Kentucky, Michigan, North Carolina, North Dakota, Ohio, South Dakota, and Tennessee). As exposure risk among children is likely dictated by the knowledge and attitudes of their caregivers, separate surveys were distributed to collect data about the adult population and the population of caregivers with children below the age of 18. In the adult survey, respondents were prompted to answer all questions for themselves, while in the caregiver survey, respondents were prompted to answer some questions for themselves and some on behalf of their child. In total, four separate surveys were deployed: two (adults and caregivers) in high-incidence jurisdictions and two (adults and caregivers) in neighboring jurisdictions. Specific survey items are detailed in Supplementary Tables S1–S4, and included questions around self-assessed knowledge of LD, attitudes toward vaccination in general and toward hypothetical future LD vaccines, and practices that might put them at high risk of exposure to ticks.

Surveys were programmed and distributed using Qualtrics online software (Qualtrics, Provo, UT) and data were collected from a panel of respondents maintained by Qualtrics. This panel provider recruits respondents through various sources, including website intercept recruitment, member referrals, targeted email lists, gaming sites, customer loyalty web portals, permission-based networks, and social media. This panel has been previously used for surveys related to many topics, including tickborne diseases (17, 18). The Qualtrics panel has been shown to provide demographically representative samples of the United States (19). Panelists received email invitations to participate in the survey; the invitations included standard language about the incentive and a hyperlink to the survey. Informed consent was obtained from all respondents before beginning the survey, and respondents were provided with information regarding the survey objectives and their right to end the survey at any time. Respondents were compensated for completing the survey using different methods (e.g., gift cards, airline points, cash). As this was a non-probability sample with an unknown sampling frame, the response rate is not reported (20).

The target sample sizes were 800 respondents for each of the four samples. Sample quotas for age, sex, race/ethnicity, and residence in a large city were used to approximate the population distribution. Respondents were considered eligible if they resided in a high-incidence or neighboring jurisdiction and were 18 years or older. Data were collected in 2023 from high-incidence jurisdictions between April 26 to May 22 and from neighboring jurisdictions between October 2 to 25. Additional methods related to survey design, administration, and recruitment procedures have been described previously (21, 22).

### 2.2 Data analysis

Data were screened to remove responses that failed data quality metrics: failed an attention check, completed the survey too quickly, or ‘straight-lined’ responses. Data were also manually checked by a researcher before analysis.

Because of sample size limitations, race was pooled into three categories for analysis: White, Black or African American, and Other. The Other category included any respondents that selected “Hispanic or Latino,” “Asian or Pacific Islander,” “Some other race/ethnicity,” or selected more than one race/ethnicity option. Analyses were conducted separately for each jurisdiction-level LD incidence category (high-incidence and neighboring) and respondent type (adults and caregivers).

Differences in responses to survey items between racial categories were assessed using Fisher’s exact test, with White respondents as the reference group. As the respondents included in the Other category represent heterogeneous identities, the results presented in the main text only compare White and Black respondents; results comparing White and Other respondents are included in the supplement. LD risk practices were quantified by assessing the number of different activities respondents reported engaging in (Supplementary Table S1C); differences in the mean number of risk activities reported were assessed using a Welch two-sample t-test.

To examine differences in knowledge about LD across high-incidence and neighboring jurisdictions, Likert scale responses to the knowledge questions were treated as a continuous dependent variable to estimate ordinary least squares regression models that controlled for urbanicity of residence. Other covariates, including income and education, were considered for inclusion in regression models but eliminated due to collinearity with urbanicity. *p*-values of <0.05 were considered statistically significant. All data analysis was conducted using R (version 4.2.2) (23).

## 3 Results

In total, the four surveys recruited 2,249 respondents who identified as White, 493 who identified as Black or African American, and 674 who identified as a race other than Black or White. The demographics of survey respondents are shown in Table 1. There were differences in respondent demographics by race across surveys in age, income, and urbanicity of residence. A higher proportion of White respondents were in the oldest age and highest income categories compared with respondents who were Black or of other races. White respondents were also more likely to report living in a small city or rural area, while Black respondents were more likely to report living in a large city.

**Table 1:** Demographic characteristics of survey respondents.

Characteristic	High incidence jurisdictions						Neighboring jurisdictions					
	Adults			Caregivers			Adults			Caregivers		
	White (*N* = 592)	Black (*N* = 119)	Other (*N* = 163)	White (*N* = 571)	Black (*N* = 114)	Other (*N* = 149)	White (*N* = 567)	Black (*N* = 131)	Other (*N* = 189)	White (*N* = 520)	Black (*N* = 129)	Other (*N* = 173)
	*n (%)*	*n (%)*	*n (%)*	*n (%)*	*n (%)*	*n (%)*	*n (%)*	*n (%)*	*n (%)*	*n (%)*	*n (%)*	*n (%)*
**Sex**						*		*	*			*
Male	267 (45%)	58 (49%)	69 (42%)	302 (53%)	61 (54%)	60 (40%)	299 (53%)	47 (36%)	75 (40%)	280 (54%)	59 (46%)	56 (32%)
Female	320 (54%)	61 (51%)	92 (56%)	267 (47%)	53 (46%)	86 (58%)	262 (46%)	84 (64%)	105 (56%)	235 (45%)	69 (53%)	112 (65%)
Non-binary/Other	5 (1%)	0 (0%)	1 (1%)	1 (0%)	0 (0%)	3 (2%)	1 (0%)	0 (0%)	4 (2%)	3 (1%)	1 (1%)	1 (1%)
Prefer not to say	0 (0%)	0 (0%)	1 (1%)	1 (0%)	0 (0%)	0 (0%)	5 (1%)	0 (0%)	5 (3%)	2 (0%)	0 (0%)	4 (2%)
**Age of child**						*					*	*
1–4 years old	–	–	–	118 (21%)	15 (13%)	20 (13%)	–	–	–	101 (19%)	38 (29%)	59 (34%)
5–10 years old	–	–	–	163 (29%)	41 (36%)	58 (39%)	–	–	–	153 (29%)	37 (29%)	57 (33%)
11–17 years old	–	–	–	290 (51%)	58 (51%)	71 (48%)	–	–	–	266 (51%)	54 (42%)	57 (33%)
**Age of respondent**		*	*		*	*		*	*		*	*
18–24 years old	29 (5%)	17 (14%)	35 (21%)	14 (3%)	2 (2%)	5 (3%)	43 (8%)	17 (13%)	48 (25%)	13 (3%)	14 (11%)	12 (7%)
25–34 years old	88 (15%)	36 (30%)	43 (26%)	157 (27%)	21 (18%)	39 (26%)	83 (15%)	33 (25%)	33 (17%)	113 (22%)	39 (30%)	69 (40%)
35–44 years old	83 (14%)	27 (23%)	37 (23%)	90 (16%)	37 (32%)	59 (40%)	110 (19%)	26 (20%)	44 (23%)	128 (25%)	30 (23%)	48 (28%)
45–54 years old	94 (16%)	23 (19%)	20 (12%)	69 (12%)	19 (17%)	35 (23%)	92 (16%)	16 (12%)	32 (17%)	44 (9%)	11 (9%)	24 (14%)
55–64 years old	92 (16%)	8 (7%)	16 (10%)	213 (37%)	27 (24%)	6 (4%)	81 (14%)	19 (15%)	17 (9%)	171 (33%)	32 (25%)	17 (10%)
65+ years old	206 (35%)	8 (7%)	12 (7%)	28 (5%)	8 (7%)	5 (3%)	158 (28%)	20 (15%)	15 (8%)	51 (10%)	3 (2%)	3 (2%)
**Education**					*	*		*				
Some high school or less	9 (2%)	0 (0%)	1 (1%)	5 (1%)	2 (2%)	1 (1%)	17 (3%)	4 (3%)	7 (4%)	20 (4%)	3 (2%)	6 (4%)
High school diploma or GED	320 (54%)	74 (62%)	78 (48%)	280 (49%)	76 (67%)	52 (35%)	371 (65%)	105 (80%)	126 (67%)	324 (62%)	93 (72%)	106 (61%)
Bachelor’s degree or more	263 (44%)	45 (38%)	83 (51%)	286 (50%)	36 (32%)	95 (64%)	172 (30%)	22 (17%)	55 (29%)	175 (34%)	32 (25%)	57 (33%)
Prefer not to say	0 (0%)	0 (0%)	1 (1%)	0 (0%)	0 (0%)	1 (1%)	7 (1%)	0 (0%)	1 (1%)	1 (2%)	1 (1%)	4 (2%)
**Income**		*	*		*			*				
$0–40,000	141 (24%)	43 (36%)	59 (36%)	104 (18%)	27 (24%)	26 (17%)	227 (40%)	80 (61%)	78 (41%)	172 (33%)	57 (44%)	54 (31%)
$ 40,001–100,000	282 (48%)	57 (48%)	58 (36%)	243 (43%)	65 (57%)	63 (42%)	253 (45%)	41 (31%)	77 (41%)	243 (47%)	51 (40%)	82 (47%)
$ 100,001 or more	146 (25%)	15 (13%)	38 (23%)	218 (38%)	22 (19%)	56 (38%)	62 (11%)	7 (5%)	21 (11%)	97 (19%)	21 (16%)	28 (16%)
Prefer not to say	23 (4%)	4 (3%)	8 (5%)	6 (1%)	0 (0%)	4 (3%)	25 (4%)	3 (2)	13 (7%)	8 (2%)	0 (0%)	9 (5%)
**Residence**		*	*		*	*		*	*		*	*
A large city	98 (17%)	53 (45%)	59 (36%)	117 (20%)	43 (38%)	44 (30%)	101 (18%)	61 (47%)	51 (27%)	103 (20%)	58 (45%)	58 (34%)
A suburb near a large city	236 (40%)	42 (35%)	60 (37%)	231 (40%)	53 (46%)	79 (53%)	193 (34%)	27 (21%)	66 (35%)	143 (28%)	31 (24%)	65 (38%)
A small city / rural area	258 (44%)	24 (20%)	44 (27%)	223 (39%)	18 (16%)	26 (17%)	273 (48%)	43 (33%)	72 (38%)	274 (53%)	40 (31%)	50 (29%)

*indicates Fisher’s exact test *p*-value <0.05, comparing White to Black or Other.

Intention to vaccinate for LD was not significantly different between respondents of different races across most survey items (Table 2; Supplementary Table S2). In neighboring states, Black adults reported a higher intention to vaccinate against LD than White adults (18% ‘Very likely’ in Black adults vs. 9% ‘Very likely’ in White adults, *p*-value <0.05); however, there was no difference in intention to vaccinate for LD if a healthcare provider recommended vaccination. In high-incidence jurisdictions, however, Black adults reported lower intention to vaccinate for LD than White individuals if vaccination was recommended by a healthcare provider (24% ‘Very likely’ in Black adults vs. 40% ‘Very likely’ in White adults, *p*-value <0.05).

**Table 2:** Attitudes toward vaccination for LD and in general in White and Black respondents.

Survey Item	High incidence jurisdictions				Neighboring jurisdictions			
	Adults		Caregivers		Adults		Caregivers	
	White (*N* = 592)	Black (*N* = 119)	White (*N* = 571)	Black (*N* = 114)	White (*N* = 567)	Black (*N* = 131)	White (*N* = 520)	Black (*N* = 129)
	*n (%)*	*n (%)*	*n (%)*	*n (%)*	*n (%)*	*n (%)*	*n (%)*	*n (%)*
**If a vaccine for Lyme disease was available, how likely would you be to get it?**						*		
Very unlikely	42 (7%)	14 (12%)	24 (4%)	10 (9%)	83 (15%)	25 (19%)	48 (9%)	12 (9%)
Unlikely	44 (7%)	10 (8%)	30 (5%)	8 (7%)	108 (19%)	18 (14%)	54 (10%)	10 (8%)
Neither likely nor unlikely	157 (27%)	33 (28%)	89 (16%)	27 (24%)	161 (28%)	28 (21%)	115 (22%)	44 (34%)
Likely	198 (33%)	43 (36%)	257 (45%)	37 (32%)	166 (29%)	37 (28%)	201 (39%)	40 (31%)
Very likely	151 (26%)	19 (16%)	171 (30%)	32 (28%)	49 (9%)	23 (18%)	102 (20%)	23 (18%)
**If your healthcare provider recommended that you get vaccinated for Lyme disease, how likely is it that you would get vaccinated?**		*						
Very unlikely	31 (5%)	11 (9%)	21 (4%)	9 (8%)	60 (11%)	18 (14%)	34 (7%)	12 (9%)
Unlikely	36 (6%)	9 (8%)	22 (4%)	6 (5%)	72 (13%)	15 (11%)	43 (8%)	15 (12%)
Neither likely nor unlikely	97 (16%)	21 (18%)	72 (13%)	13 (11%)	113 (20%)	17 (13%)	80 (15%)	24 (19%)
Likely	190 (32%)	49 (41%)	218 (38%)	45 (39%)	208 (37%)	47 (36%)	211 (41%)	50 (39%)
Very likely	238 (40%)	29 (24%)	238 (42%)	41 (36%)	114 (20%)	34 (26%)	152 (29%)	28 (22%)
**Disagree with: I feel safe after being vaccinated**	51 (9%)	17 (14%)	47 (8%)	9 (8%)	96 (17%)	21 (16%)	74 (14%)	16 (12%)
**Disagree with: I feel protected after getting vaccinated**	46 (8%)	17 (14%) *	46 (8%)	8 (7%)	102 (18%)	19 (15%)	76 (15%)	15 (12%)
**Agree with: Although most vaccines appear to be safe, there may be problems that we have not yet discovered.**	399 (67%)	83 (70%)	391 (68%)	77 (68%)	399 (70%)	85 (65%)	371 (71%)	80 (62%) *
**Agree with: Vaccines can cause unforeseen problems in children.**	239 (40%)	56 (47%)	293 (51%)	52 (46%)	277 (49%)	62 (47%)	270 (52%)	65 (50%)
**Agree with: Vaccination programs are a big con**.	79 (13%)	29 (24%) *	115 (20%)	24 (21%)	114 (20%)	40 (31%) *	131 (25%)	42 (33%)
**Agree with: Authorities promote vaccination for financial gain, not for people’s health**	128 (22%)	35 (29%)	146 (26%)	30 (26%)	162 (29%)	38 (29%)	175 (34%)	49 (38%)

*indicates Fisher’s exact test *p*-value <0.05, comparing White to Black.

General attitudes toward vaccination were mostly similar across racial categories in each survey (Table 2; Supplementary Table S2). When differences in attitudes did exist, the proportion of Black respondents reporting concerns about vaccination was between 6 and 11% higher than that of White respondents.

In all surveys and across all outdoor environment types, a higher proportion of White respondents than Black respondents reported spending more time outdoors, though this difference was not always significant (Table 3). The trends were less consistent for respondents of other races (Supplementary Table S3). In addition, White respondents reported doing a significantly higher number of outdoor activities than Black respondents on average. White caregivers in high-incidence jurisdictions and both adults and caregivers in neighboring states were also more likely to report having a household pet that goes outside. On the other hand, in high-incidence jurisdictions, a higher proportion of Black adults (19%) and adults of other races (11%) reported having occupations that were primarily outdoor work than White adults (8%). In neighboring states, a higher proportion of adults of other races (12%) and Black caregivers (19%) reported doing primarily outdoor work compared with White adults (9%) and caregivers (17%).

**Table 3:** Practices related to LD in White and Black respondents.

Survey item	High incidence jurisdictions				Neighboring jurisdictions			
	Adults		Caregivers		Adults		Caregivers	
	White (*N* = 592)	Black (*N* = 119)	White (*N* = 571)	Black (*N* = 114)	White (*N* = 567)	Black (*N* = 131)	White (*N* = 520)	Black (*N* = 129)
	*n (%)*	*n (%)*	*n (%)*	*n (%)*	*n (%)*	*n (%)*	*n (%)*	*n (%)*
**How often do you/your child spend time outside in …**								
*Deep woods; brush; un-mowed field; or marshland*				*				*
Daily—Several Times a Week	62 (10%)	8 (7%)	105 (18%)	12 (11%)	60 (11%)	12 (9%)	101 (19%)	13 (10%)
A Few Times a Month—Every Few Months	196 (33%)	30 (25%)	230 (40%)	30 (26%)	133 (23%)	20 (15%)	166 (32%)	32 (25%)
Once or twice per year—Rarely or Never	334 (56%)	81 (68%)	236 (41%)	72 (63%)	374 (66%)	99 (76%)	253 (49%)	84 (65%)
*Wooded area with trails; mowed fields; natural yard (e.g., non-maintained grass)*		*		*		*		*
Daily—Several Times a Week	96 (16%)	16 (13%)	163 (29%)	19 (17%)	81 (14%)	11 (8%)	133 (26%)	23 (18%)
A Few Times a Month—Every Few Months	241 (41%)	34 (29%)	268 (47%)	30 (26%)	191 (34%)	35 (27%)	190 (37%)	30 (23%)
Once or twice per year—Rarely or Never	255 (43%)	69 (58%)	140 (25%)	65 (57%)	295 (52%)	85 (65%)	197 (38%)	76 (59%)
*Well-maintained yard; park; playground*		*				*		*
Daily—Several Times a Week	346 (58%)	43 (36%)	414 (73%)	73 (64%)	284 (50%)	47 (36%)	329 (63%)	59 (46%)
A Few Times a Month—Every Few Months	186 (31%)	48 (40%)	132 (23%)	32 (28%)	191 (34%)	40 (31%)	138 (27%)	55 (43%)
Once or twice per year—Rarely or Never	60 (10%)	28 (24%)	25 (4%)	9 (8%)	92 (16%)	44 (34%)	53 (10%)	15 (12%)
*Paved sidewalks; roads; porches or patios*		*				*		*
Daily—Several Times a Week	484 (82%)	88 (74%)	476 (83%)	89 (78%)	439 (77%)	88 (67%)	402 (77%)	81 (63%)
A Few Times a Month—Every Few Months	89 (15%)	17 (14%)	78 (14%)	20 (18%)	87 (15%)	24 (18%)	82 (16%)	31 (24%)
Once or twice per year—Rarely or Never	19 (3%)	14 (12%)	17 (3%)	5 (4%)	41 (7%)	19 (15%)	36 (7%)	17 (13%)
**Mean number of activities performed (SD)**	3.3 (1.0)	2.7 (1.3) ^	3.6 (0.8)	3.1 (1.0) ^	3.1 (1.1)	2.5 (1.3) ^	3.3 (1.0)	2.9 (1.1) ^
*Please indicate if your occupation is primarily indoor or outdoor work*		*						*
Not currently employed	261 (44%)	32 (27%)	101 (18%)	23 (20%)	255 (45%)	53 (40%)	149 (29%)	22 (17%)
Primarily indoor work	284 (48%)	64 (54%)	380 (67%)	67 (59%)	262 (46%)	67 (51%)	283 (54%)	83 (64%)
Primarily outdoor work (e.g., construction, landscaping, forestry, land surveying, farming, railroad/utility work)	47 (8%)	23 (19%)	90 (16%)	24 (21%)	50 (9%)	11 (8%)	88 (17%)	24 (19%)
**Household has a dog that goes outside**	216 (36%)	49 (41%)	376 (66%)	48 (42%) *	256 (45%)	38 (29%) *	356 (68%)	53 (41%) *
**Household has a cat that goes outside**	84 (14%)	11 (9%)	160 (28%)	16 (14%) *	105 (19%)	16 (12%)	159 (31%)	27 (21%) *

*indicates Fisher’s exact test *p*-value <0.05, comparing White to Black. ^indicates Welch two sample *t*-test *p*-value <0.05, comparing White to Black.

In all surveys, unadjusted knowledge of LD for Black respondents was significantly lower than for White respondents (Table 4). After adjusting for urbanicity of residence and jurisdiction incidence level, compared with White respondents, self-assessed knowledge of LD in both the adult (Table 5, Model 1) and caregiver (Table 5, Model 2) samples was significantly lower for Black respondents (0.29 in adults, 0.30 in caregivers) and respondents of other races (0.14 in adults, 0.32 in caregivers). However, perception of LD as a common or serious problem was not significantly associated with race (Table 5, Models 3–6).

**Table 4:** Knowledge of LD in White and Black respondents.

Survey item	High incidence jurisdictions				Neighboring jurisdictions			
	Adults		Caregivers		Adults		Caregivers	
	White (*N* = 592)	Black (*N* = 119)	White (*N* = 571)	Black (*N* = 114)	White (*N* = 567)	Black (*N* = 131)	White (*N* = 520)	Black (*N* = 129)
	*n (%)*	*n (%)*	*n (%)*	*n (%)*	*n (%)*	*n (%)*	*n (%)*	*n (%)*
**How much do you know about Lyme disease?**		*		*		*		*
None	36 (6%)	21 (18%)	16 (3%)	11 (10%)	79 (14%)	46 (35%)	31 (6%)	38 (29%)
A little	280 (47%)	57 (48%)	211 (37%)	45 (39%)	305 (54%)	56 (43%)	255 (49%)	53 (41%)
Some	212 (36%)	32 (27%)	250 (44%)	52 (46%)	156 (28%)	24 (18%)	174 (33%)	33 (26%)
A lot	64 (11%)	9 (8%)	94 (16%)	6 (5%)	27 (5%)	5 (4%)	60 (12%)	5 (4%)
**How common do you think Lyme disease is in the community where you live?**		*		*		*		*
Rare	133 (22%)	46 (39%)	105 (18%)	44 (39%)	263 (46%)	66 (50%)	210 (40%)	49 (38%)
Somewhat common	188 (32%)	31 (26%)	166 (29%)	20 (18%)	152 (27%)	24 (18%)	144 (28%)	26 (20%)
Common	128 (22%)	15 (13%)	171 (30%)	23 (20%)	56 (10%)	8 (6%)	68 (13%)	19 (15%)
Very Common	87 (15%)	8 (7%)	92 (16%)	5 (4%)	20 (4%)	4 (3%)	40 (8%)	5 (4%)
Do not know	56 (10%)	19 (16%)	37 (7%)	22 (19%)	76 (13%)	29 (22%)	58 (11%)	30 (23%)
**How serious a problem would you say Lyme disease is in your community?**		*		*		*		*
Not a problem at all	48 (8%)	20 (17%)	40 (7%)	15 (13%)	117 (21%)	32 (24%)	94 (18%)	21 (16%)
Not much of a problem	207 (35%)	44 (37%)	183 (32%)	38 (33%)	244 (43%)	38 (29%)	194 (37%)	40 (31%)
Somewhat serious problem	198 (33%)	21 (18%)	231 (40%)	32 (28%)	91 (16%)	15 (11%)	115 (22%)	25 (19%)
Very serious problem	60 (10%)	14 (12%)	77 (13%)	11 (10%)	33 (6%)	13 (10%)	63 (12%)	18 (14%)
Do not know	79 (13%)	20 (17%)	40 (7%)	18 (16%)	82 (14%)	33 (25%)	54 (10%)	25 (19%)

*indicates Fisher’s exact test *p*-value <0.05, comparing White to Black.

**Table 5:** Linear regression models of knowledge by race, adjusted for residence.

Model number	Knowledge question (1–4 scale)	Respondent type	Intercept	Race coefficient (White REF)		Residence coefficient (Large City REF)		State incidence (HighREF)	Race: neighboring interaction (White REF)		Residence: neighboring interaction (Large City REF)	
				Black or AA	Other	Suburb	Small city / rural	Neighboring	Black or AA	Other	Suburb	Small city / rural
1	How much do you know about Lyme disease? ^	Adults	2.56	−0.29 *	−0.14 *	−0.03	−0.08	−0.26 *	−0.06	0.01	−0.07	0.00
2		Caregivers	2.80	−0.30 *	−0.32 *	−0.14	−0.03	−0.17 *	−0.20	−0.01	−0.03	−0.11
3	How common do you think Lyme disease is in the community where you live? ^^	Adults	2.47	−0.17	−0.05	0.02	0.19	−0.40 *	0.35	0.15	−0.08	−0.08
4		Caregivers	2.65	−0.12	−0.03	−0.09	−0.13	−0.41 *	0.44 *	0.21	−0.12	0.10
5	How serious a problem would you say Lyme disease is in your community? ^^^	Adults	2.73	−0.06	0.13	0.11	0.19	−0.23	0.38 *	0.24	−0.11	−0.17
6		Caregivers	2.88	0.00	0.12	−0.05	−0.12	−0.17	0.26	−0.16	−0.14	0.06

*indicates *p*-value <0.05. ^1 = None, 2 = A little, 3 = Some, 4 = A lot; ^^1 = Rare, 2 = Somewhat common, 3 = Common, 4 = Very Common; ^^^1 = Not a problem at all, 2 = Not much of a problem, 3 = Somewhat serious problem, 4 = Very serious problem.

After adjusting for race and jurisdiction incidence level, urbanicity of residence was not associated with LD knowledge (Table 5). Self-assessed knowledge for respondents in neighboring states was lower by 0.26 for adults and 0.17 for caregivers (Table 5, Model 1–2). Respondents from neighboring states were also less likely to think LD was common in their area (Table 5, Model 3–4), but did not have significantly different perceptions about the seriousness of LD (Table 5, Model 5–6). There were no differences in relationships between knowledge and race and knowledge and residence in high-incidence vs. neighboring jurisdictions, with two exceptions: Black caregivers in neighboring states were more likely to think of LD as common than White caregivers (Table 5, Model 4), and Black adults in neighboring states were more likely to say LD was a serious problem in their community than White adults (Table 5, Model 5).

## 4 Discussion

This study provides insights into how self-assessed knowledge, attitudes toward vaccines for LD and in general, and risk practices for LD compare between persons in different racial categories in jurisdictions with a high incidence of LD and neighboring jurisdictions. While these data show that there are differences in some measures between racial groups, findings differed based on geography (i.e., high-incidence jurisdiction vs. neighboring jurisdiction) and survey population (adult vs. caregiver). Overall, these findings suggest that White respondents had more knowledge about LD, less concerns about vaccination in general, and a higher frequency of time spent outdoors. The only exception to this is occupational risk, with Black respondents being more likely to report an outdoor occupation.

Our sample included both adults and caregivers of children under 18, allowing insight into some of the factors influencing LD knowledge and exposure practices among children as well. LD incidence has been shown to peak in children aged 5–9 years old (24), and children from racial and ethnic minority groups are more likely to have disseminated LD manifestations than their white counterparts (9). Our findings were concurrent across both the adult and caregiver samples, which emphasizes the consistency of these disparities across both adult and child populations. This highlights the critical need to address these issues through targeted interventions that address both adult and pediatric needs.

White respondents reported more frequent participation in recreational outdoor risk practices (e.g., hiking in wooded areas, walking around public parks) than Black respondents, which could be explained by a higher proportion of Black respondents living in urban areas and thus being less likely to have access to recreational green spaces. This is further compounded by racial disparities in access to green spaces within US cities, as having a higher proportions of White residents have been shown to correlate with greater access to urban green spaces (25).

Higher knowledge and more frequent participation in outdoor risk practices among White respondents could be related: public health education around LD may be targeted toward individuals spending recreational time outside. Though Black respondents were more likely to have outdoor occupations, it is possible that LD preventative messaging is not effectively reaching those exposed in an occupational capacity. Further, though White respondents were more likely to live in rural areas, self-assessed knowledge by racial group still differed after adjustment for urbanicity of residence. This suggests that discrepancies in knowledge between racial categories cannot be solely explained by the differences in where individuals of different races live and highlights a need to better shape messages to ensure that they reach all at-risk individuals regardless of race. On the other hand, White respondents may be more likely to have experience with LD, either personally or through someone close to them, as reported incidence of LD is higher in White individuals (6–9). As such, higher knowledge in White respondents could reflect greater experience with the disease. Future studies could investigate sources of information about LD, the degree of trust in those sources, and how these might vary by race.

Given the paucity of literature on racial differences in LD, comparing these findings with existing evidence is difficult. Two previous studies in Delaware and New York found that White individuals were more likely to perceive LD as a problem (10, 11). While the unadjusted analyses from this study concur with the findings from previous studies, adjusting for urbanicity rendered this difference insignificant in high-incidence jurisdictions. In neighboring states, however, Black adults were more likely than White adults to perceive LD as being a serious problem in their community, even after adjusting for urbanicity.

In addition, two other studies also found higher willingness to receive a prospective vaccine to prevent LD in White respondents compared with adults from racial or ethnic minority groups (12) and Black, non-Hispanic adults (14). This difference in intention to receive a hypothetical vaccine by race has also been found for non-LD diseases (14). It is worth noting that respondents were asked about their intention to receive a vaccine not yet available in both these studies and this study; responses may not necessarily hold in the context of an available vaccine. Still, there is a substantial body of literature on racial differences in uptake of recommended adult and childhood vaccines, which generally finds lower uptake among people from racial or ethnic minority groups (26–28). This relationship is highly complex and mediated by many other socioeconomic factors; in particular, Black Americans have been found to report lower trust in the medical system and vaccine approval process (28), a sentiment rooted in the historic mistreatment of Black Americans in the medical system (29).

Though the findings of this study agree with existing evidence on the perception of LD as a problem and intention to receive an LD vaccine, this study as a whole offers additional information beyond existing literature surrounding racial differences in LD. While other studies have focused on measuring and comparing knowledge, attitudes, and practices across high-incidence and neighboring jurisdictions, this is the first focused examination of how these parameters differ by race (18). The inclusion of equity in the Evidence to Recommendations Framework put forth by the Advisory Committee on Immunization Practices (ACIP) underscores the importance of considering issues such as knowledge gaps or vaccine acceptability that may vary between groups and impede equitable vaccine access and usage (30).

A major limitation of these findings is the ability to conduct analyses for specific race and ethnicity groups representing a relatively smaller proportion of the US population. Though the survey was powered to approximate the demographic distribution of these jurisdictions, the data remain underpowered to adequately analyze persons identifying as races other than Black or White. As respondents in the Other category are likely heterogeneous, it is difficult to draw meaningful conclusions about the ethnic or racial groups included in that category without intentionally oversampling to provide the power necessary for analyses. In particular, persons of Hispanic origin were not broken out as a separate category in this study, but there is research suggesting that there are disparities in disease severity and higher occupational risk among Hispanic persons (31). Further, race is a construct experienced across multiple, at times conflicting, dimensions (32). Respondents in this study were asked to self-identify as a particular race, but this may not capture other aspects of race that can drive differences in lived experiences (e.g., skin color, first language, immigration status). Though we can describe some differences in knowledge between racial categories that are independent of differences in urbanicity, it is difficult to tease out how this arises.

Another limitation is the comparison of four different surveys: adults in high-incidence jurisdictions, caregivers in high-incidence jurisdictions, adults in neighboring jurisdictions, and caregivers in neighboring jurisdictions. Though most questions were the same across all surveys (Supplementary Tables S1A–D), caregivers were prompted in some questions to answer questions on behalf of their child and in other questions to answer for themselves. In addition, the surveys in high-incidence jurisdictions were fielded before or early in the tick season while the surveys in neighboring states were fielded after or late in the tick season. As a result, it is possible that respondents in neighboring states had more recent exposure to ticks or public awareness campaigns about tickborne diseases. Thus, these data are not directly compared between surveys.

## 5 Conclusion

This study shows that there are some racial differences in knowledge, attitudes, and risk practices related to LD, with White respondents generally reporting higher knowledge, less concerns about vaccination, and a higher frequency of time spent outdoors. That these differences are not fully explained by differences in urbanicity suggests that LD knowledge and exposure practices may partly underlie the disparities in LD outcomes. However, the complexity of LD risk requires further research to understand the mechanisms by which these differences are created and maintained.

## Data Availability

The datasets presented in this article are not readily available due to respondent privacy. Requests to access the datasets should be directed to madiha.shafquat@pfizer.com.
