# Supplementary material for: Racial differences in knowledge, attitudes toward vaccination, and risk practices around Lyme disease in the United States
Source: Front Public Health. 2025 Mar 21;13:1473304. doi: 10.3389/fpubh.2025.1473304 (PMC11970133; doi:10.3389/fpubh.2025.1473304)
Supplement: Supplementary file 1 [file Data_Sheet_1.docx]

| **Table S1A. Demographic questions included in surveys** | | | |
| --- | --- | --- | --- |
| **Construct** | **Items** | **Scale** | **Notes** |
| Race | Which one or more of the following would you use to describe yourself? | 1 = White, 2 = Black or African American, 3 = Hispanic or Latino, 4 = Asian or Pacific Islander, 5 = Some other race/ethnicity |  |
| Sex | How do you identify? | 1 = Male, 2 = Female, 3 = Nonbinary, 4 = Prefer not to answer |  |
| Age of respondent | How old are you? | 1 = Under 18 years old, 2 = 18-24 years old, 3 = 25-34 years old, 4 = 35-44 years old, 5 = 45-54 years old, 6 = 55-64 years old, 7 = 65+ years old |  |
| Age of child | How old is your child? | 1 = 1-4 years old, 2 = 5-10 years old, 3 = 11-17 years old | Only shown in Caregiver survey; Caregiver only able to respond on behalf of a single child |
| Education | What is the highest grade or year of school you have completed? | 1 = Some high school or less, 2 = High school diploma or GED, 3 = Some college, but no degree, 4 = Associates or technical degree, 5 = Bachelor’s degree, 6 = Graduate or professional degree (MA, MS, MBA, PhD, JD, MD, DDS etc.), 7 = Prefer not to say |  |
| Income | What is your approximate total household income (before taxes)? | 1 = $0 – 20,000, 2 = $ 20,001 – 40,000, 3 = $ 40,001 – 60,000, 4 = $ 60,001 – 80,000, 5 = $ 80,001 – 100,000, 6 = $ 100,001 – 150,000, 7 = $ 150,001 – 200,000, 8 = $ 200,001 – 250,000, 9 = $ 250,001 or more, 10 = Prefer not to say |  |
| Residence | Which of the following best describes the place where you now live? | 1 = A large city, 2 = A suburb near a large city, 3 = A small city or town, 4 = A rural area |  |

| **Table S1B. Attitude questions included in surveys** | | | |
| --- | --- | --- | --- |
| **Construct** | **Items** | **Scale** | **Notes** |
| Vaccine intention | If a vaccine for Lyme disease was available, how likely would you be to [get it // get your child vaccinated]? | 1 = Very unlikely, 2 = Unlikely, 3 = Neither likely nor unlikely, 4 = Likely, 5 = Very likely | Caregivers asked to respond on behalf of their child |
|  | If [your // your child’s] healthcare provider recommended that [you // they] get vaccinated for Lyme disease, how likely [is it that you would get vaccinated // would you be to get your child vaccinated]? | 1 = Very unlikely, 2 = Unlikely, 3 = Neither likely nor unlikely, 4 = Likely, 5 = Very likely | Caregivers asked to respond on behalf of their child |
| Vaccine attitudes | Please rate your agreement with the following statements:   - I feel safe after being vaccinated - I feel protected after getting vaccinated - Although most vaccines appear to be safe, there may be problems that we have not yet discovered - Vaccines can cause unforeseen problems in children - Vaccination programs are a big con - Authorities promote vaccination for financial gain, not for people’s health | 1 = Strongly disagree, 2 = Disagree, 3 = Neither agree nor disagree, 4 = Agree, 5 = Strongly agree |  |

| **Table S1C. Practice questions included in surveys** | | | |
| --- | --- | --- | --- |
| **Construct** | **Items** | **Scale** | **Notes** |
| Duration | How often [do you // does your child] spend time outside in each of the following environments?   - Deep woods; brush; un-mowed field; or marshland - Wooded area with trails; mowed fields; natural yard (e.g., non-maintained grass) - Well-maintained yard; park; playground - Paved sidewalks; roads; porches or patios | 1 = Daily, 2 = Several Times a Week, 3 = A Few Times a Month, 4 = Every Few Months, 5 = Once or twice per year, 6 = Rarely or Never | Caregivers asked to respond on behalf of their child |
| Occupation | Please indicate if your occupation is primarily outdoor work or indoor work. | 1 = Primarily outdoor work (e.g., construction, landscaping, forestry, land surveying, farming, railroad/utility work), 2 = Primarily indoor work, 3 = Not currently employed |  |
| Dog ownership | Does your household have a dog that goes outside? | 1 = Yes, 2 = No |  |
| Cat ownership | Does your household have a cat that goes outside? | 1 = Yes, 2 = No |  |

| **Table S1D. Knowledge questions included in surveys** | | |
| --- | --- | --- |
| **Items** | **Scale** | **Notes** |
| How much do you know about Lyme disease? | 1 = None, 2 = A little, 3 = Some, 4 = A lot |  |
| How common do you think Lyme disease is in the community where you live? | 1 = Rare, 2 = Somewhat common, 3 = Common, 4 = Very Common |  |
| How serious a problem would you say Lyme disease is in your community? | 1 = Not a problem at all, 2 = Not much of a problem, 3 = Somewhat serious problem, 4 = Very serious problem |  |

**Table S2: Attitudes towards vaccination for LD and in general in respondents of Other races.** * indicates Fisher’s exact test p-value <0.05, comparing White to Other.

|  | **High incidence jurisdictions** | | **Neighboring jurisdictions** | |
| --- | --- | --- | --- | --- |
|  | **Adults (N = 163)** | **Caregivers (N = 149)** | **Adults (N = 189)** | **Caregivers (N = 173)** |
| **Survey Item** | *n*  *(%)* | *n*  *(%)* | *n*  *(%)* | *n*  *(%)* |
| **If a vaccine for Lyme disease was available, how likely would you be to get it?** |  | ***** |  |  |
| Very unlikely | 10  (6%) | 15 (10%) | 20 (11%) | 16  (9%) |
| Unlikely | 8  (5%) | 6  (4%) | 25 (13%) | 14  (8%) |
| Neither likely nor unlikely | 34 (21%) | 37 (25%) | 50 (26%) | 50 (29%) |
| Likely | 70 (43%) | 43 (29%) | 72 (38%) | 71 (41%) |
| Very likely | 41 (25%) | 48 (32%) | 22 (12%) | 22 (13%) |
| **If your healthcare provider recommended that you get vaccinated for Lyme disease, how likely is it that you would get vaccinated?** |  |  |  |  |
| Very unlikely | 6  (4%) | 11  (7%) | 17  (9%) | 14  (8%) |
| Unlikely | 8  (5%) | 6  (4%) | 17  (9%) | 7  (4%) |
| Neither likely nor unlikely | 25 (15%) | 25 (17%) | 40 (21%) | 38 (22%) |
| Likely | 60 (37%) | 50 (34%) | 74 (39%) | 70 (40%) |
| Very likely | 64 (39%) | 57 (38%) | 41 (22%) | 44 (25%) |
| **Disagree with: I feel safe after being vaccinated** | 16 (10%) | 17 (11%) | 21 (11%) | 24 (14%) |
| **Disagree with: I feel protected after getting vaccinated** | 14  (9%) | 15 (10%) | 19 (10%) * | 22 (13%) |
| **Agree with: Although most vaccines appear to be safe, there may be problems that we have not yet discovered.** | 97 (60%) | 95 (64%) | 127 (67%) | 113 (65%) |
| **Agree with: Vaccines can cause unforeseen problems in children.** | 68 (42%) | 70 (47%) | 81 (43%) | 97 (56%) |
| **Agree with: Vaccination programs are a big con.** | 26 (16%) | 30 (20%) | 43 (23%) | 40 (23%) |
| **Agree with: Authorities promote vaccination for financial gain, not for people’s health** | 40 (25%) | 40 (27%) | 57 (30%) | 66 (38%) |

**Table S3. Practices related to LD in respondents of Other races.*** indicates Fisher’s exact test p-value <0.05, comparing White to Other. ^ indicates Welch two sample t-test p-value <0.05, comparing White to Other.

|  | **High incidence jurisdictions** | | **Neighboring jurisdictions** | |
| --- | --- | --- | --- | --- |
|  | **Adults (N = 163)** | **Caregivers (N = 149)** | **Adults (N = 189)** | **Caregivers (N = 173)** |
| **Survey Item** | *n*  *(%)* | *n*  *(%)* | *n*  *(%)* | *n*  *(%)* |
| **How often do you/your child spend time outside in …** | | | | |
| *Deep woods; brush; un-mowed field; or marshland* |  |  |  |  |
| Daily - Several Times a Week | 16 (10%) | 21 (14%) | 26 (14%) | 26 (15%) |
| A Few Times a Month - Every Few Months | 46 (28%) | 51 (34%) | 54 (29%) | 58 (34%) |
| Once or twice per year - Rarely or Never | 101 (62%) | 77 (52%) | 109 (58%) | 89 (51%) |
| *Wooded area with trails; mowed fields; natural yard (e.g., non-maintained grass)* |  | ***** |  |  |
| Daily - Several Times a Week | 26 (16%) | 27 (18%) | 35 (19%) | 34 (20%) |
| A Few Times a Month - Every Few Months | 64 (39%) | 61 (41%) | 66 (35%) | 60 (35%) |
| Once or twice per year - Rarely or Never | 73 (45%) | 61 (41%) | 88 (47%) | 79 (46%) |
| *Well-maintained yard; park; playground* | ***** | ***** |  |  |
| Daily - Several Times a Week | 69 (42%) | 85 (57%) | 94 (50%) | 101 (58%) |
| A Few Times a Month - Every Few Months | 63 (39%) | 52 (35%) | 67 (35%) | 59 (34%) |
| Once or twice per year - Rarely or Never | 31 (19%) | 12  (8%) | 28 (15%) | 13  (8%) |
| *Paved sidewalks; roads; porches or patios* | ***** |  |  |  |
| Daily - Several Times a Week | 123 (75%) | 114 (77%) | 134 (71%) | 137 (79%) |
| A Few Times a Month - Every Few Months | 26 (16%) | 27 (18%) | 42 (22%) | 30 (17%) |
| Once or twice per year - Rarely or Never | 14  (9%) | 8  (5%) | 13  (7%) | 6  (4%) |
| **Mean number of activities performed (SD)** | 3.2 (1.1) | 3.4 (0.9) | 3.1 (1.1) | 3.2 (0.9) |
| **Please indicate if your occupation is primarily indoor or outdoor work** | ***** |  | ***** |  |
| Not currently employed | 52 (32%) | 31 (21%) | 57 (30%) | 38 (22%) |
| Primarily indoor work | 93 (57%) | 102 (68%) | 110 (58%) | 112 (65%) |
| Primarily outdoor work (e.g., construction, landscaping, forestry, land surveying, farming, railroad/utility work) | 18 (11%) | 16 (11%) | 22 (12%) | 23 (13%) |
| **Household has a dog that goes outside** | 60 (37%) | 85 (57%) | 88 (47%) | 103 (60%) * |
| **Household has a cat that goes outside** | 16 (10%) | 32 (21%) | 36 (19%) | 37 (21%) * |

**Table S4. Knowledge of LD in respondents of Other races.** * indicates Fisher’s exact test p-value <0.05, comparing White to Other.

|  | **High incidence jurisdictions** | | **Neighboring jurisdictions** | |
| --- | --- | --- | --- | --- |
|  | **Adults (N = 163)** | **Caregivers (N = 149)** | **Adults (N = 189)** | **Caregivers (N = 173)** |
| **Survey Item** | *n*  *(%)* | *n*  *(%)* | *n*  *(%)* | *n*  *(%)* |
| **How much do you know about Lyme disease?** |  | ***** |  | ***** |
| None | 19 (12%) | 14  (9%) | 38 (20%) | 27 (16%) |
| A little | 76 (47%) | 68 (46%) | 101 (53%) | 92 (53%) |
| Some | 54 (33%) | 54 (36%) | 42 (22%) | 46 (27%) |
| A lot | 14  (9%) | 13  (9%) | 8  (4%) | 8  (5%) |
| **How common do you think Lyme disease is in the community where you live?** | ***** | ***** |  |  |
| Rare | 56 (34%) | 46 (31%) | 92 (49%) | 71 (41%) |
| Somewhat common | 39 (24%) | 32 (21%) | 38 (20%) | 39 (23%) |
| Common | 28 (17%) | 30 (20%) | 22 (12%) | 18 (10%) |
| Very Common | 13  (8%) | 22 (15%) | 4  (2%) | 13  (8%) |
| Don't know | 27 (17%) | 19 (13%) | 33 (17%) | 32 (18%) |
| **How serious a problem would you say Lyme disease is in your community?** |  |  | ***** |  |
| Not a problem at all | 18 (11%) | 15 (10%) | 34 (18%) | 43 (25%) |
| Not much of a problem | 52 (32%) | 42 (28%) | 64 (34%) | 53 (31%) |
| Somewhat serious problem | 44 (27%) | 49 (33%) | 33 (17%) | 32 (18%) |
| Very serious problem | 18 (11%) | 24 (16%) | 9  (5%) | 21 (12%) |
| Don't know | 31 (19%) | 19 (13%) | 49 (26%) | 24 (14%) |
